# Enhancement of Inflammatory Protein Expression and Nuclear Factor Κb (NF-Κb) Activity by Trichostatin A (TSA) in OP9 Preadipocytes

**DOI:** 10.1371/journal.pone.0059702

**Published:** 2013-03-26

**Authors:** Taiki Sato, Daisuke Kotake, Masahiro Hiratsuka, Noriyasu Hirasawa

**Affiliations:** Laboratory of Pharmacotherapy of Life-Style Related Diseases, Graduate School of Pharmaceutical Sciences, Tohoku University, Sendai, Japan; Charité-Universitätsmedizin Berlin, Germany

## Abstract

The production of inflammatory proteins such as interleukin-6 (IL-6) by preadipocytes and mature adipocytes is closely associated with the impairment of systemic glucose homeostasis. However, precisely how the production is regulated and the roles of histone deacetylases (HDACs) remain largely unknown. The aim of this study was to establish whether HDAC inhibitors affect the expression of inflammatory proteins in pre/mature adipocytes, and, if so, to determine the mechanism involved. Trichostatin A (TSA), an HDAC inhibitor, enhanced lipopolysaccharide (LPS)-induced production of IL-6 in OP9 preadipocytes but not the mature adipocytes. Moreover, TSA also enhanced palmitic acid-induced IL-6 production and the expression of inflammatory genes induced by LPS in preadipocytes. Although TSA did not affect TLR4 mRNA expression or the activation of MAPKs, a reporter gene assay revealed that the LPS-induced increase in nuclear factor κB (NF-κB) activity was enhanced by TSA. Moreover, TSA increased the level of NF-κB p65 acetylation at lysine 310 and duration of its translocation into the nucleus, which leads to enhancement of NF-κB activity and subsequently expression of inflammatory genes. These findings shed new light on the regulatory roles of HDACs in preadipocytes in the production of inflammatory proteins.

## Introduction

Chronic inflammation in adipose tissue is closely associated with the impairment of systemic glucose homeostasis [1]. In fact, preadipocytes and mature adipocytes produce inflammatory cytokines and chemokines such as interleukin-6 (IL-6) and monocyte chemoattractant protein-1 (MCP-1), respectively, on stimulation of toll-like receptor 4 (TLR4). IL-6 impairs glucose-responsive insulin secretion in islets [2]. Moreover, circulating IL-6 causes insulin resistance by increasing suppressor of cytokine signaling 3 expression in hepatocytes and mature adipocytes *in vivo* and *in vitro* [3]–[7]. MCP-1 induces infiltration of macrophages into adipose tissue, which exacerbates inflammation and the subsequent impairment of glucose homeostasis [8].

Lipopolysaccharide (LPS), an abundant glycolipid of the outer membrane of gram-negative bacteria, is recognized by TLR4 and induces inflammatory responses in various cells [9], [10]. A small amount of LPS is constitutively absorbed through the gut even in a healthy state. This triggers weak systemic inflammation including in adipose tissue, which, in turn, leads to impairment of glucose homeostasis, which is called metabolic endotoxemia [11]. Moreover, saturated free fatty acids including palmitic acid (PA) also induce inflammatory responses as ligands of TLR4 [12], [13]. TLR4 signaling triggers the activation of transcription factors including nuclear factor κB (NF-κB) and of mitogen-activated protein kinases (MAPKs) such as extracellular signal-regulated kinase (ERK), p38 MAPK and c-Jun N-terminal kinase (JNK) [9]. NF-κB is activated via phosphorylation and degradation of IκB, an inhibitory factor of NF-κB, by the IκB kinase (IKK) enzyme complex [14], [15], which leads to the nuclear translocation of NF-κB and the subsequent transcription of NF-κB-dependent genes, such as the IL-6, MCP-1, cyclooxygenase-2 (COX-2) and inducible nitric oxide synthase (iNOS) genes.

Histone deacetylases (HDACs) regulate the expression of various genes by removing acetyl tags of histones and non-histone proteins, including transcription factors. Conversely, histone acetyltransferases (HATs) competitively add acetyl tags to these proteins [16]–[18]. The modification of proteins by acetylation leads to changes in functions. Thus, HDACs and HATs play important roles in the regulation of gene expression. To date, 18 members of the HDAC family have been identified [19]. The class I (HDACs1, 2, 3 and 8) and class II (HDACs4, 5, 6, 7, 9, 10 and 11) isoforms are Zn-dependent, whereas class III HDACs (Sirtuins1−7) are NAD^+^-dependent. Trichostatin A (TSA, IC50 = 1−10 nM) and suberoylanilide hydroxamic acid (SAHA, IC50 = 10−300 nM) have a hydroxamate structure and inhibit Zn-dependent HDAC subtypes (HDAC1−11) broadly [19], [20].

It has been shown that HDACs are closely involved in inflammatory responses and HDAC inhibitors affect the expression of inflammatory proteins in various cell lines. For example, TSA enhanced inflammatory gene transcription in microglial cells, epithelial cells, fibroblasts, thymocytes and splenocytes [21]–[24]. It also did the same in peripheral blood mononuclear cells from diabetics [25]. Additionally, knockdown of HDAC2 by siRNA enhanced LPS-induced granulocyte macrophage-colony stimulating factor expression in epithelial cells and primary human lung macrophages [26]. On the other hand, TSA repressed IL-1β/LPS/IFNγ-induced iNOS expression in murine macrophages [27]. However, the roles of HDACs in the inflammatory responses of preadipocytes and mature adipocytes, which are important in the maintenance of systemic glucose homeostasis, remain largely unknown. We therefore examined the effects of HDAC inhibitors on the expression of inflammatory proteins in pre/mature adipocytes.

## Materials and Methods

### Reagents

Insulin from bovine pancreas, oleic acid, troglitazone (TGZ) and TPCA-1 were purchased from Sigma-Aldrich (St. Louis, MO). TSA, LPS and PA were from Wako Chemical Co. (Osaka, Japan). SAHA was from Cayman Chemical (Ann Arbor, MI). Tumor necrosis factor alpha (TNFα) was from R&D Systems (Minneapolis, MN). U0126 was purchased from Promega (Madison, WI). SB203580 was from Calbiochem (San Diego, CA). SP600125 was from Biomol Research Laboratories (Plymouth Meeting, PA). Insulin from bovine pancreas was dissolved in 0.02 N HCl-sterile water. TGZ, U0126, SB203580, SP600125 and TPCA-1 were dissolved in dimethyl sulfoxide. TSA and SAHA were dissolved in ethanol. LPS was dissolved in sterile water. PA was dissolved in 2% (w/v) BSA-ethanol. TNFα was dissolved in 0.1% (w/v) BSA-PBS.

### Cell culture and drug treatments

OP9 cells (obtained from Riken Bio Resource Center Cell Bank (RCB1124), Tsukuba, Japan), a line of bone marrow-derived mouse stromal cells, were cultured in minimum essential medium alpha (MEM-α, Invitrogen, Carlsbad, CA) supplemented with 20% (v/v) heat-inactivated fetal bovine serum (FBS, Biowest, Miami, FL), 18 µg/ml penicillin G potassium (Meiji Seika, Tokyo, Japan) and 50 µg/ml streptomycin sulfate (Meiji Seika) at 37°C in a humidified atmosphere of 95% (v/v) air and 5% (v/v) CO_2_. The cells were seeded in each well of a multi well plate (Becton, Dickinson and Company, Franklin Lakes, NJ) at 5,000 cells/cm^2^. 3T3-L1 cells (obtained from Health Science Research Resources Bank, Japanese Collection of Research Bioresources Cell Bank (JCRB9014), Osaka, Japan) were cultured in Dulbecco's modified Eagle's medium (Nissui Seiyaku, Tokyo, Japan) supplemented with 10% (v/v) FBS, 18 µg/ml penicillin G potassium and 50 µg/ml streptomycin sulfate at 37°C in a humidified atmosphere of 95% (v/v) air and 5% (v/v) CO_2_. The cells were seeded in each well of a multi well plate at 15,000 cells/cm^2^. Both OP9 cells and 3T3-L1 cells were incubated at 37°C for 3 days until confluent, and then used for experiments. They were preincubated for 1 hour in the presence or absence of HDAC inhibitors (TSA and SAHA), MAPK inhibitors (U0126, SB203580, and SP600125) or an IKK-2 inhibitor (TPCA-1), and stimulated with LPS, PA or TNFα for a specified period of time.

### Differentiation into mature adipocytes

**Figure 1:**
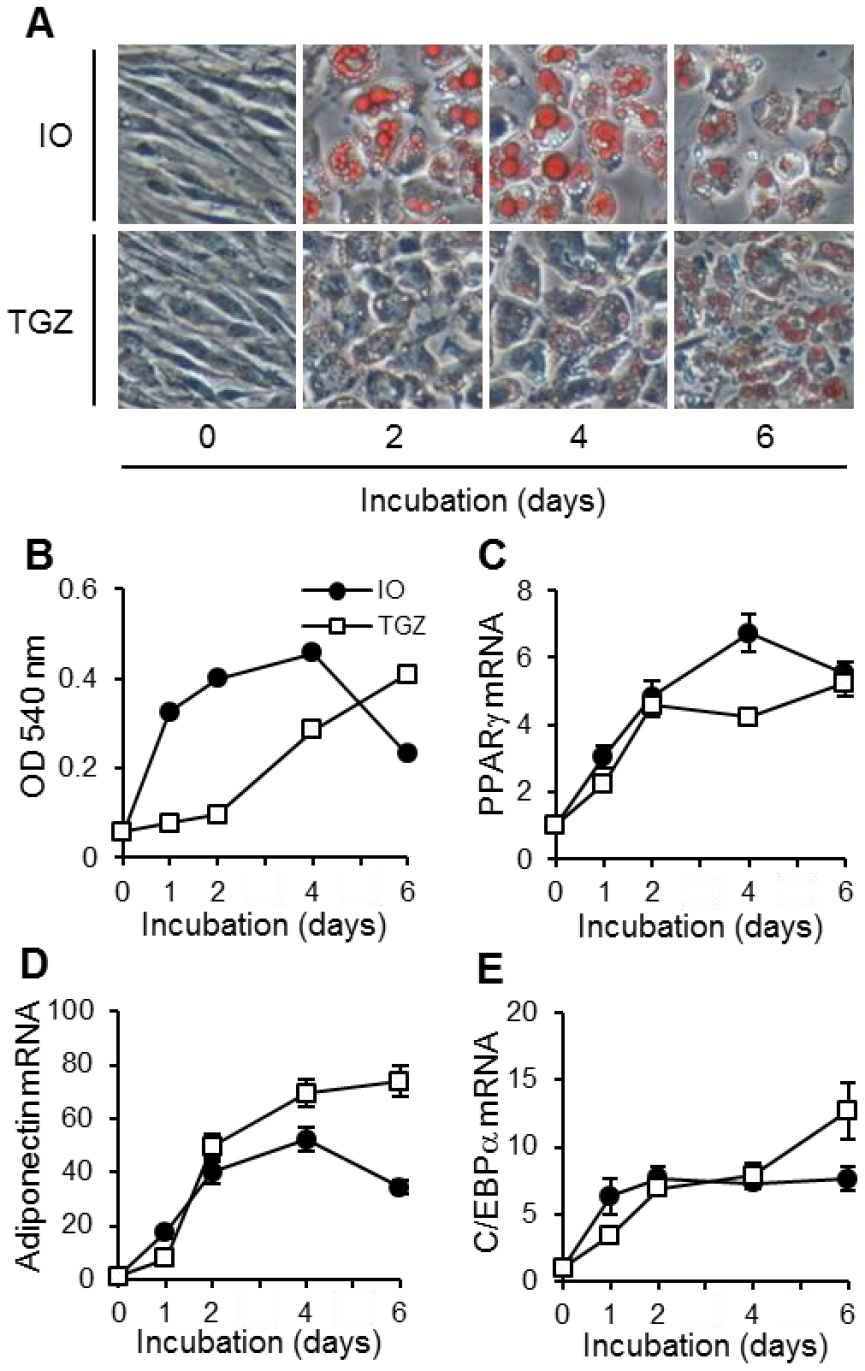
Differentiation of OP9 preadipocytes into mature adipocytes. OP9 cells were incubated in medium containing IO or TGZ for the periods indicated. The cells were stained with Oil red O and observed under a phase-contrast microscope (A). The lipid contents of OP9 cells were determined by measuring the absorbance of Oil red O re-solubilized with isopropanol at 540 nm (B). The levels of mRNA for PPARγ (C), adiponectin (D) and C/EBPα (E) were determined using real-time PCR. Values are normalized to those of 18 S rRNA and the mean value at time 0 is set to 1.0. Data represent the mean ± S.E.M. (*n* = 4).

Adipocyte differentiation induced by insulin and oleic acid (IO) was performed according to the method described by Wolins et al. [28] with minor modifications. Briefly, OP9 cells were seeded at 5,000 cells/cm^2^ and grown in MEM-α supplemented with 20% (v/v) FBS, 18 µg/ml penicillin G potassium and 50 µg/ml streptomycin sulfate for 3 days until confluent (day 0, preadipocytes) and then cultured for specified periods in MEM-α with 0.2% (v/v) FBS, 175 nM insulin and 900 µM oleic acid bound to albumin (5.5∶1 molar ratio). Adipocyte differentiation was induced by TGZ as follows: OP9 cells were seeded at 5,000 cells/cm^2^, grown for 3 days until confluent (day 0, preadipocytes), and treated for specified periods with MEM-α supplemented with 20% (v/v) FBS and 3 µM TGZ. Typical morphological aspects of OP9 preadipocytes and differentiated adipocytes are represented in Figure 1A.

### Oil red O staining

Cells were washed with phosphate-buffered saline (PBS), then fixed in 10% (v/v) neutralized formalin (pH 7.2) for 10 minutes at room temperature. After a wash with PBS, 60% (v/v) isopropanol was added for 1 minute. Then, cells were stained in a 0.18% (w/v) Oil red O solution (Sigma-Aldrich) for 15 minutes at room temperature. The cells were washed with 60% (v/v) isopropanol and PBS, and observed under a phase-contrast microscope (Nikon, Tokyo, Japan). For quantification of lipid contents, the Oil red O in the cells was extracted with 100% isopropanol for 15 minutes at room temperature and the absorbance of extracts at 540 nm was determined.

### ELISA

IL-6 and MCP-1 levels were measured by ELISA according to the manufacturer's instructions (eBioscience, San Diego, CA, and R&D systems, respectively).

### Quantitative real-time PCR

Total RNA was extracted using a GenElute mammalian total RNA kit (Sigma-Aldrich). cDNA was synthesized from 500 ng of the total RNA by reverse transcription using PrimeScript RT master mix (Takara, Shiga, Japan) according to the manufacturer's instructions, and diluted 10 fold with Tris-EDTA buffer. Real-time PCR was performed using SYBR premix Ex Taq II (Takara) in a Thermal cycler dice real time system (Takara). As an internal control, levels of glyceraldehyde-3-phosphate dehydrogenase (GAPDH) mRNA or 18 S ribosomal RNA (18 S rRNA) were quantified in parallel with target genes. The primers used for real-time PCR were (forward) 5′-ACTTCGGAATCAGCTCTGTG-3′ and (reverse) 5′-TCCATAGTGGAAGCCTGATG-3′ for mouse peroxisome proliferator-activated receptor gamma (PPARγ), (forward) 5′-CTCTTAATCCTGCCCAGTCA-3′ and (reverse) 5′-TACACATAAGCGGCTTCTCC-3′ for mouse adiponectin, (forward) 5′-GGACTCTGATCATGGCACTG-3′ and (reverse) 5′-CTGATCCATGCATTGGTAGGT-3′ for mouse TLR4 [29], (forward) 5′-AGTTGCCTTCTTGGGACTGA-3′ and (reverse) 5′-CAGAATTGCCATTGCACAAC-3′ for mouse IL-6 [30], (forward) 5′-GAAGTCTTTGGTCTGGTGCCTG-3′ and (reverse) 5′-GTCTGCTGGTTTGGAATAGTTGC-3′ for mouse COX-2 [31], (forward) 5′-GGAGCGAGTTGTGGATTGTC-3′ and (reverse) 5′-GTGAGGGCTTGGCTGAGTGAG-3′ for mouse iNOS [31], (forward) 5′-CCTGTCATGCTTCTGGGCCTGC-3′ and (reverse) 5′-GGGGCGTTAACTGCATCTGGCTG-3′ for mouse MCP-1, (forward) 5′-TTGACGGAAGGGCACCACCAG-3′ and (reverse) 5′-GCACCACCACCCACGGAATCG-3′ for mouse 18 S rRNA [32] and (forward) 5′-TGTGTCCGTCGTGGATCTGA-3′ and (reverse) 5′-TTGCTGTTGAAGTCGCAGGAG-3′ for mouse GAPDH. Normalization and fold changes were calculated using the ΔΔCt method.

### Western blotting

Western blotting was carried out as described previously [33]. The antibodies used as the primary antibody were a rabbit anti-acetyl-histone H4 (Lys8) antibody (Santa Cruz Biotechnology, Santa Cruz, CA), a rabbit anti-histone H4 antibody (Santa Cruz Biotechnology), a goat anti-actin antibody (Santa Cruz Biotechnology), a rabbit anti-phospho-p44/p42 (ERK1/2) MAPK (Thr202/Thr204) antibody (Cell Signaling Technology, Beverly, MA), a rabbit anti-p44/p42 (ERK1/2) MAPK antibody (Upstate Biotechnology, Lake Placid, NY), a rabbit anti-phospho-p38 MAPK (Thr180/Thr182) antibody, a rabbit anti-p38 MAPK antibody (Santa Cruz Biotechnology), a rabbit anti-acetyl-NF-κB p65 (Lys310) antibody and a rabbit anti-NF-κB p65 antibody (Santa Cruz Biotechnology). Those used as the secondary antibody were a biotinylated anti-rabbit IgG (Vector Laboratories, Burlingame, CA), a horseradish peroxidase-conjugated anti-rabbit IgG (Cell Signaling Technology) and a horseradish peroxidase-conjugated anti-goat IgG (Santa Cruz Biotechnology).

### Plasmid constructs and site-directed mutagenesis

The NF-κB-responsive luciferase reporter gene was obtained from Stratagene (La Jolla, CA), and the *Renilla* luciferase gene (control, pGL4.75[*hRluc*/CMV] Vector), from Promega. The murine IL-6 promoter (−1232 to +39) was supplied by Dr. T. Kishimoto, Osaka University, Japan and Dr. A. Kimura, Keio University, Japan, and integrated into the pGL3-basic vector (Promega). Mutations in the putative NF-κB, AP-1 and CCAAT/enhancer binding protein (C/EBP)-binding sites or cAMP response element (CRE) in the murine IL-6 promoter were generated using the QuikChange lightning site-directed mutagenesis kit (Stratagene) according to the manufacturer's instructions. The primers used for site-directed mutagenesis were (forward) 5′-CAAATGTGGGATTTTAGACTGAGTCTCAAAATTAGAGAG-3′ and (reverse) 5′-CTCTCTAATTTTGAGACTCAGTCTAAAATCCCACATTTG-3′ for mutations of the NF-κB-binding site [34], (forward) 5′- GTGGGATTTTCCCATGCAGCTCAAAATTAGAGAGTTG-3′ and (reverse) 5′- CAACTCTCTAATTTTGAGCTGCATGGGAAAATCCCAC-3′ for mutations of the upstream AP-1-binding site [34], (forward) 5′-TCCCATCAAGACATGCTCAAGTGCTGCAGCACTTTTAAAGAAAAAAAAGAAGAGTG-3′ and (reverse) 5′-CACTCTTCTTTTTTTTCTTTAAAAGTGCTGCAGCACTTGAGCATGTCTTGATGGGA-3′ for mutations of the downstream AP-1-binding site, (forward) 5′-CGATGCTAAACGACGTCACAGATATCAATCTTAATAAGG-3′ and (reverse) 5′-CCTTATTAAGATTGATATCTGTGACGTCGTTTAGCATCG-3′ for mutations of the C/EBP-binding site [34], and (forward) 5′-CCTAGTTGTGATTCTTTCGATGCTAAACGGATCCACATTGTGCAATCTTAATAAGGTTTCCA-3′ and (reverse) 5′-TGGAAACCTTATTAAGATTGCACAATGTGGATCCGTTTAGCATCGAAAGAATCACAACTAGG-3′ for mutations of CRE. Mutations were verified by sequencing.

### Luciferase assay

OP9 cells were transfected in 24-well plates with the target (500 ng) and control (20 ng) luciferase reporter plasmid in 48 µl of Opti-MEM (Invitrogen), per well, using X-tremeGENE HP DNA transfection reagent (Roche, Basel, Switzerland) according to the manufacturer's instructions. After 24 hours, the culture medium was changed and the cells were stimulated. Luciferase activity in the cells after 8 hours of LPS stimulation was determined using a Dual-luciferase reporter assay system (Promega).

### Immunostaining

After stimulation, OP9 cells on 96-well plates (Becton, Dickinson and Company) were fixed in 4% neutral buffered paraformaldehyde for 10 minutes and then −20°C methanol for 10 minutes. The cells were next permeated in 0.5% (v/v) Triton X-100-PBS for 10 minutes. They were blocked in 4% (w/v) Block Ace (DS Pharma Biomedical, Osaka, Japan) for 1 hour at room temperature, and incubated overnight at 4°C with the rabbit anti-NF-κB p65 antibody. After being washed with PBS containing 0.1% (v/v) Tween20, the cells were incubated at room temperature with the Alexa Fluor 488-conjugated anti-rabbit IgG antibody (Invitrogen). Then, they were stained with 300 nM 4′,6-diamidino-2-phenylindole (DAPI) for 5 minutes at room temperature, and fluorescence images of 4 randomly chosen fields were taken using an IN Cell Analyzer 2000 (GE Healthcare, Buckinghamshire, UK). NF-κB nuclear translocation was calculated with an established protocol using IN Cell WorkStation software (GE Healthcare).

### Fractionation of nucleus and cytoplasm

Fractionation of the nucleus and cytoplasm was performed using a nuclear extract kit (Active Motif, Carlsbad, CA) according to the manufacturer's instructions.

### Statistical analysis

The statistical significance of the results was analyzed using Student's t-test or a one-way ANOVA with the Bonferroni/dunnett post hoc test for multiple comparisons.

## Results

### OP9 preadipocytes were made to differentiate into mature adipocytes using both methods

As shown in Fig. 1A, treatments both with IO and with TGZ caused the formation of many lipid droplets in the cytosol of OP9 cells. The lipid contents reached a maximum at 4 days with the IO-based method and at 6 days with the TGZ-based method (Fig. 1B). Moreover, the mRNA expression of adipocyte differentiation markers such as PPARγ (Fig. 1C), adiponectin (Fig. 1D) and C/EBPα (Fig. 1E) was increased by the treatments.

### TSA did not affect TLR4-mediated IL-6 production in mature adipocytes

**Figure 2:**
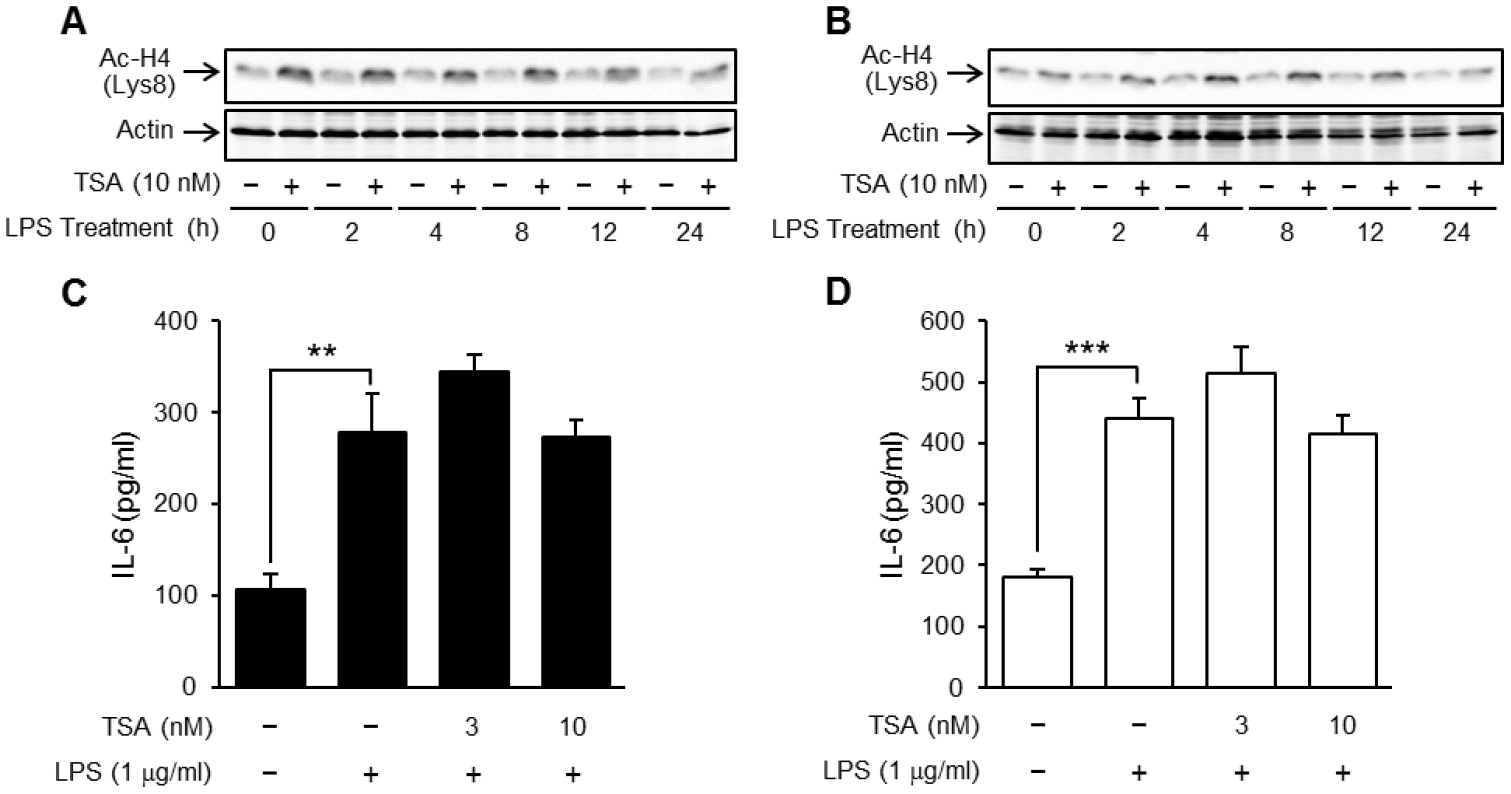
Effects of TSA on TLR4-mediated IL-6 production in mature adipocytes. Mature OP9 adipocytes induced to differentiate by IO for 4 days (A, C) or TGZ for 6 days (B, D) were preincubated for 1 hour in the presence or absence of TSA, and then stimulated with LPS (1 μg/ml) for the period indicated. The acetylated histone H4 was detected by Western blotting (A, B). The concentration of IL-6 in the medium 24 hours after the stimulation was determined by ELISA (C, D). Data represent the mean ± S.E.M. (n = 4). ***P<0.001, **P<0.01 between the indicated groups.

IO and TGZ-treated mature OP9 adipocytes were incubated for 1 hour in the presence or absence of TSA (3 and 10 nM), and then stimulated with LPS (1 µg/ml). The acetylation levels of histone H4 were apparently increased by TSA (10 nM) during the pretreatment for 1 hour (at time 0) and lasted 24 hours in both differentiated cells (Fig. 2A and B). On the other hand, the stimulation by LPS caused the production of IL-6 in mature adipocytes but was not affected by pretreatment with TSA (3 and 10 nM) (Fig. 2C and D).

### TSA enhanced TLR4-mediated IL-6 production and inflammatory gene expression in preadipocytes

**Figure 3:**
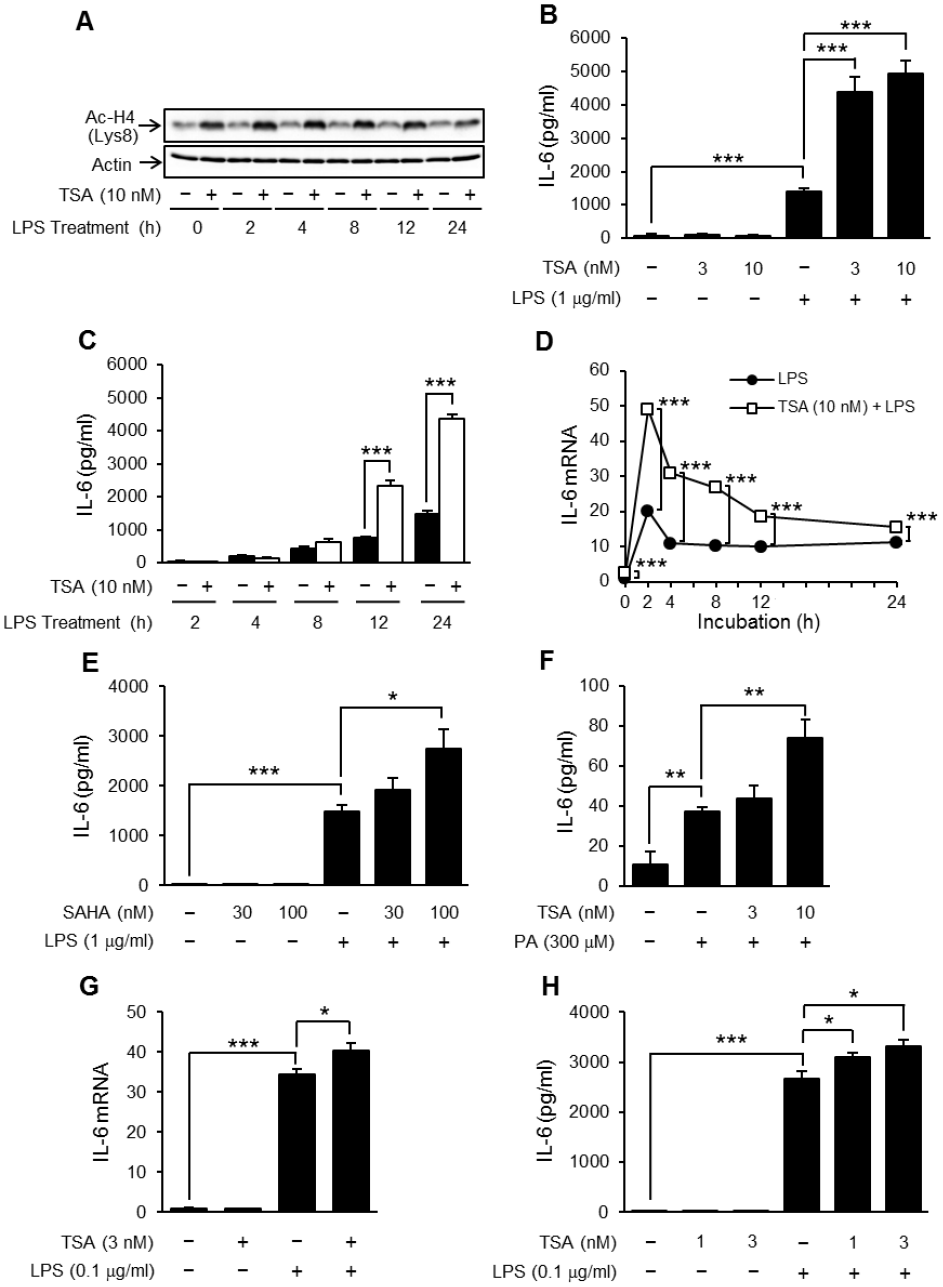
Effects of TSA on TLR4-mediated IL-6 production in preadipocytes. OP9 preadipocytes (A−F) and 3T3-L1 preadipocytes (G, H) were preincubated for 1 hour in the presence or absence of TSA (A−E, G, H) or SAHA (F), and then stimulated with LPS (A−D, F−H) or PA (300 µM) (E). The acetylated histone H4 was detected by Western blotting (A). The concentration of IL-6 in the medium 24 hours (B, E, F, H) or at the indicated time (C) after the stimulation was determined by ELISA. The levels of mRNA for IL-6 at the indicated time (D) or 2 hours (G) after the stimulation were determined using real-time PCR. Values are normalized to those of GAPDH mRNA and the mean value at time 0 (LPS alone) is set to 1.0. Data represent the mean ± S.E.M. (*n* = 4). ***P<0.001, **P<0.01, *P<0.05 between the indicated groups.

Next we carried out similar experiments in OP9 preadipocytes. As in the differentiated adipocytes, TSA increased acetylation levels of histone H4 during 1 hour of pretreatment (at time 0) and the effect lasted 24 hours (Fig. 3A). However, unlike in the mature adipocytes, TSA significantly enhanced the LPS-induced IL-6 production although TSA alone did not induce IL-6 production (Fig. 3B). This enhancing effect of TSA appeared from 12 hours after LPS stimulation (Fig. 3C). Levels of IL-6 mRNA in LPS-stimulated OP9 cells increased within 2 hours and remained high for 24 hours. TSA also significantly increased the levels of IL-6 mRNA from 2 to 24 hours after LPS stimulation (Fig. 3D). Moreover, treatment with PA (300 µM) induced IL-6 production, which was also significantly enhanced by TSA pretreatment (Fig. 3E). SAHA (100 nM), another pan-HDAC inhibitor, enhanced LPS-induced IL-6 production as well (Fig. 3F). Similar results were obtained with 3T3-L1 cells, another preadipocyte cell line. Namely, TSA enhanced LPS-induced IL-6 production significantly at both the mRNA (Fig. 3G) and protein (Fig. 3H) levels.

**Figure 4:**
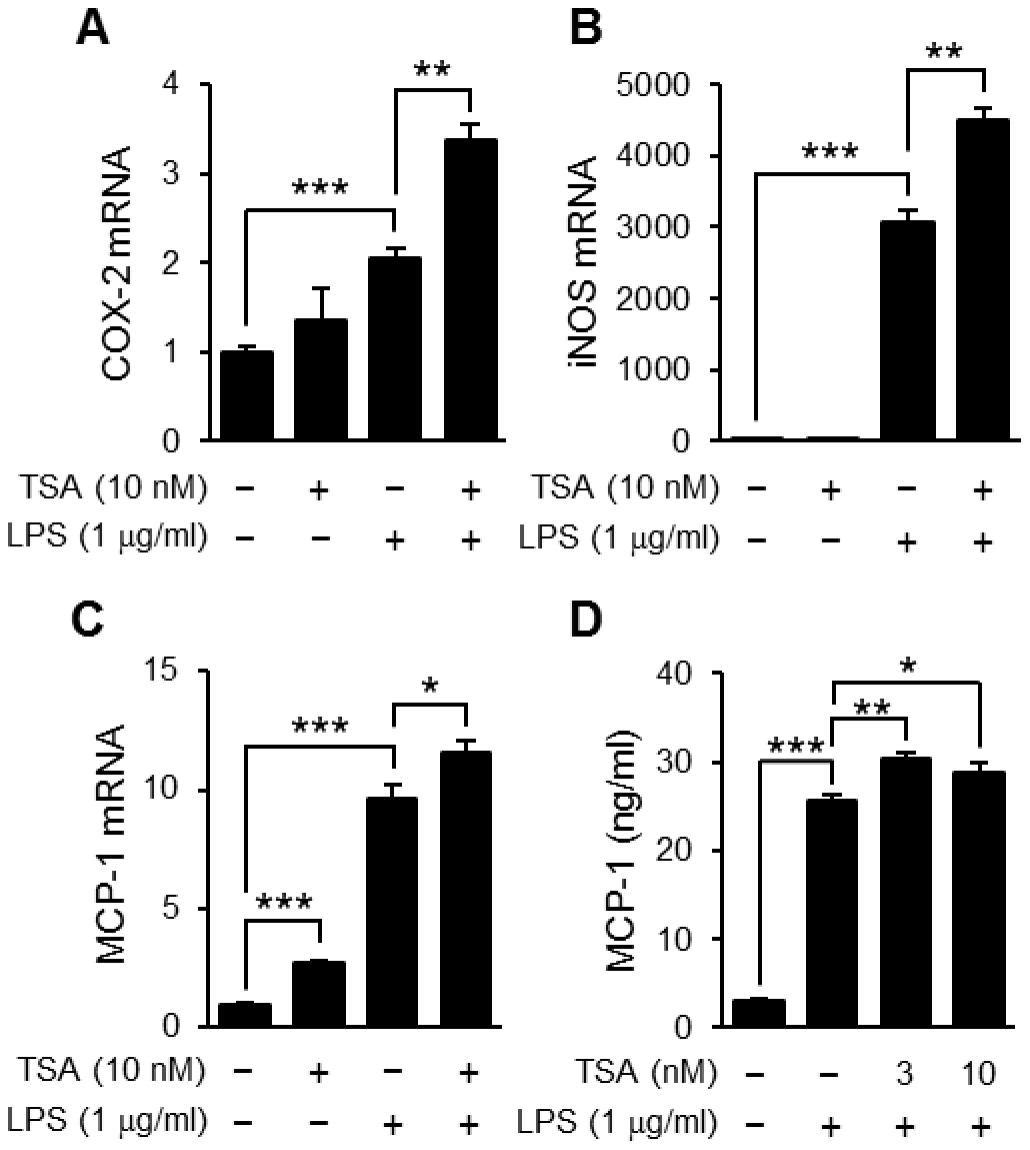
Effects of TSA on the expression of TLR4-mediated inflammatory proteins. OP9 preadipocytes were preincubated for 1 hour in the presence or absence of TSA, and then stimulated with LPS (1 µg/ml) for 2 h (A−C) or 24 h (D). The levels of mRNA for COX-2 (A), iNOS (B), and MCP-1 (C) in the cells were determined using real-time PCR. Values are normalized to those of GAPDH mRNA and the mean value for the control is set to 1.0. The concentration of MCP-1 in the medium 24 hours after the stimulation was determined by ELISA (D). Data represent the mean ± S.E.M. (*n* = 4). ***P<0.001, **P<0.01, *P<0.05 between the indicated groups.

Then, we examined whether TSA enhanced the expression of other inflammatory genes such as the COX-2, iNOS and MCP-1 genes. The expression levels of these genes were increased by LPS and all were enhanced by TSA although the effect varied (Fig. 4A, B and C). Corresponding to the level of MCP-1 mRNA, the concentration of MCP-1 in culture supernatants was increased by LPS stimulation, which was enhanced slightly but significantly by TSA pretreatment (Fig. 4D).

### The effect on inflammatory protein expression by TSA was independent of TLR4

**Figure 5:**
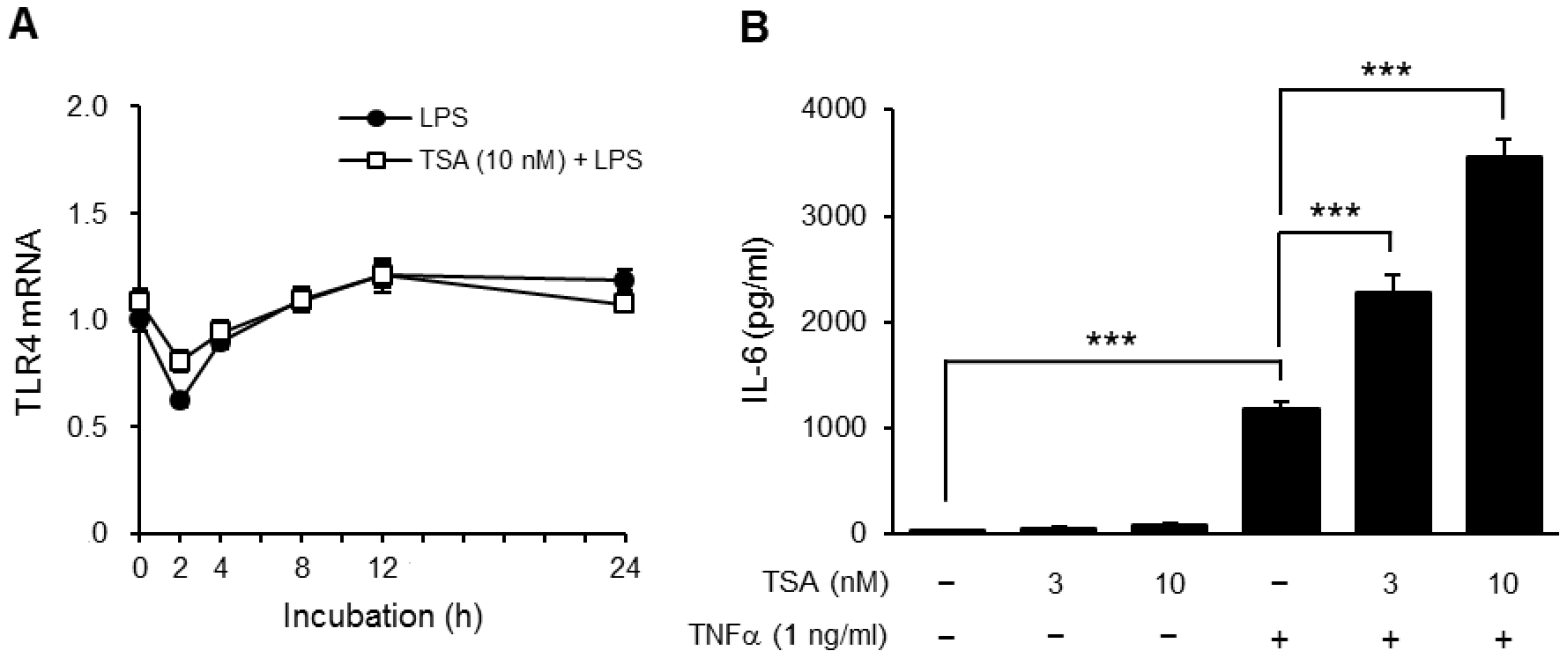
Effects of TSA on TLR4 expression and TNFα-induced IL-6 production. OP9 preadipocytes were preincubated for 1 hour in the presence or absence of TSA, and then stimulated with LPS (1 µg/ml) (A) or TNFα (1 ng/ml) (B) for the period indicated. The levels of mRNA for TLR4 were determined using real-time PCR (A). Values are normalized to those of GAPDH mRNA and the mean value at time 0 (LPS alone) is set to 1.0. The concentration of IL-6 in the medium 8 hours after the stimulation was determined by ELISA (B). Data represent the mean ± S.E.M. (*n* = 4). ***P<0.001 between the indicated groups.

To clarify the mechanism by which HDAC inhibitors enhanced the expression of inflammatory proteins in preadipocytes, we firstly examined whether TSA affected the expression of TLR4 and TLR4-independent responses. OP9 cells were incubated for 2−24 hours in medium containing LPS with or without TSA, and then mRNA levels of TLR4 were determined. The levels were slightly changed by LPS but the changes were less affected by TSA (Fig. 5A). Furthermore, TNF-α-induced IL-6 production was also significantly enhanced by TSA in OP9 preadipocytes (Fig. 5B).

### TSA did not affect LPS-induced phosphorylation of ERK1/2 and p38 MAPK

**Figure 6:**
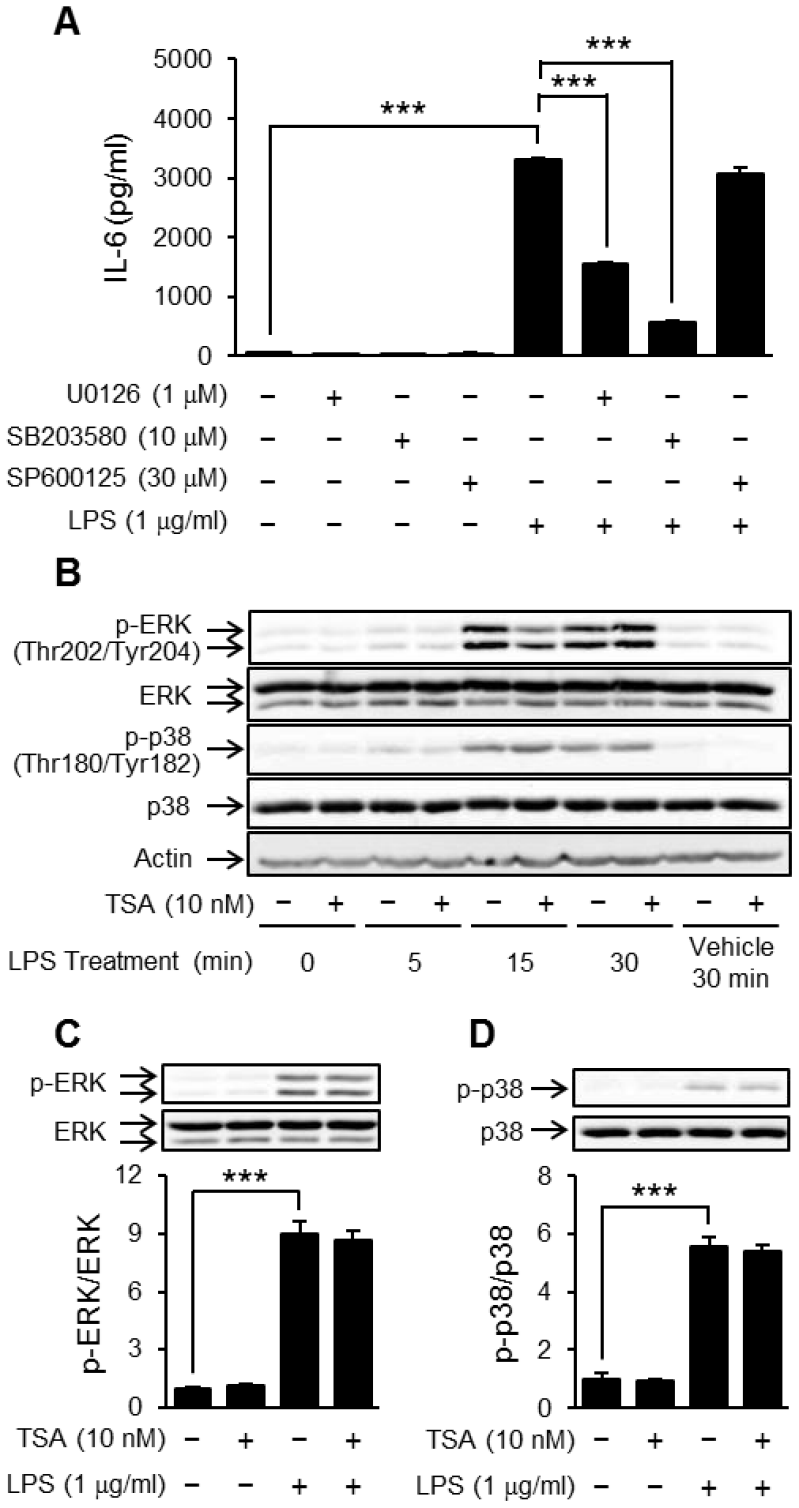
Effects of TSA on LPS-induced phosphorylation of ERK1/2 and p38 MAPK. OP9 preadipocytes were preincubated for 1 hour in the presence or absence of U0126 (1 µM), SB203580 (10 µM) or SP600125 (30 µM), and then stimulated with LPS (1 µg/ml). The concentration of IL-6 in the medium 8 hours after the stimulation was determined by ELISA (A). Data represent the mean ± S.E.M. (*n* = 3). OP9 preadipocytes were preincubated for 1 hour in the presence or absence of TSA (10 nM), and then stimulated with LPS (1 µg/ml) for the period indicated (B) or 15 minutes (C, D). The phosphorylation of ERK1/2 (B, C) and p38 MAPK (B, D) were determined with Western blotting. Data represent the mean ± S.E.M. (*n* = 4). ***P<0.001 between the indicated groups.

Secondly, we examined whether TSA augmented the LPS-induced activation of MAPKs. As shown in Fig. 6A, LPS-induced IL-6 production was significantly inhibited by U0126, an inhibitor of MAPK kinase, and by SB203580, an inhibitor of p38 MAPK, whereas it was not reduced by SP600125, an inhibitor of JNK. Therefore, we focused on LPS-induced phosphorylation of ERK1/2, downstream of MAPK kinase, and p38 MAPK. LPS induced the phosphorylation of ERK1/2 and p38 MAPK within 5 minutes and this lasted at least 30 minutes (Fig. 6B), whereas TSA affected the LPS-induced phosphorylation of neither ERK1/2 (Fig. 6C) nor p38 MAPK (Fig. 6D) at 15 minutes.

### TSA enhanced LPS-induced NF-κB-dependent transcriptional activation

**Figure 7:**
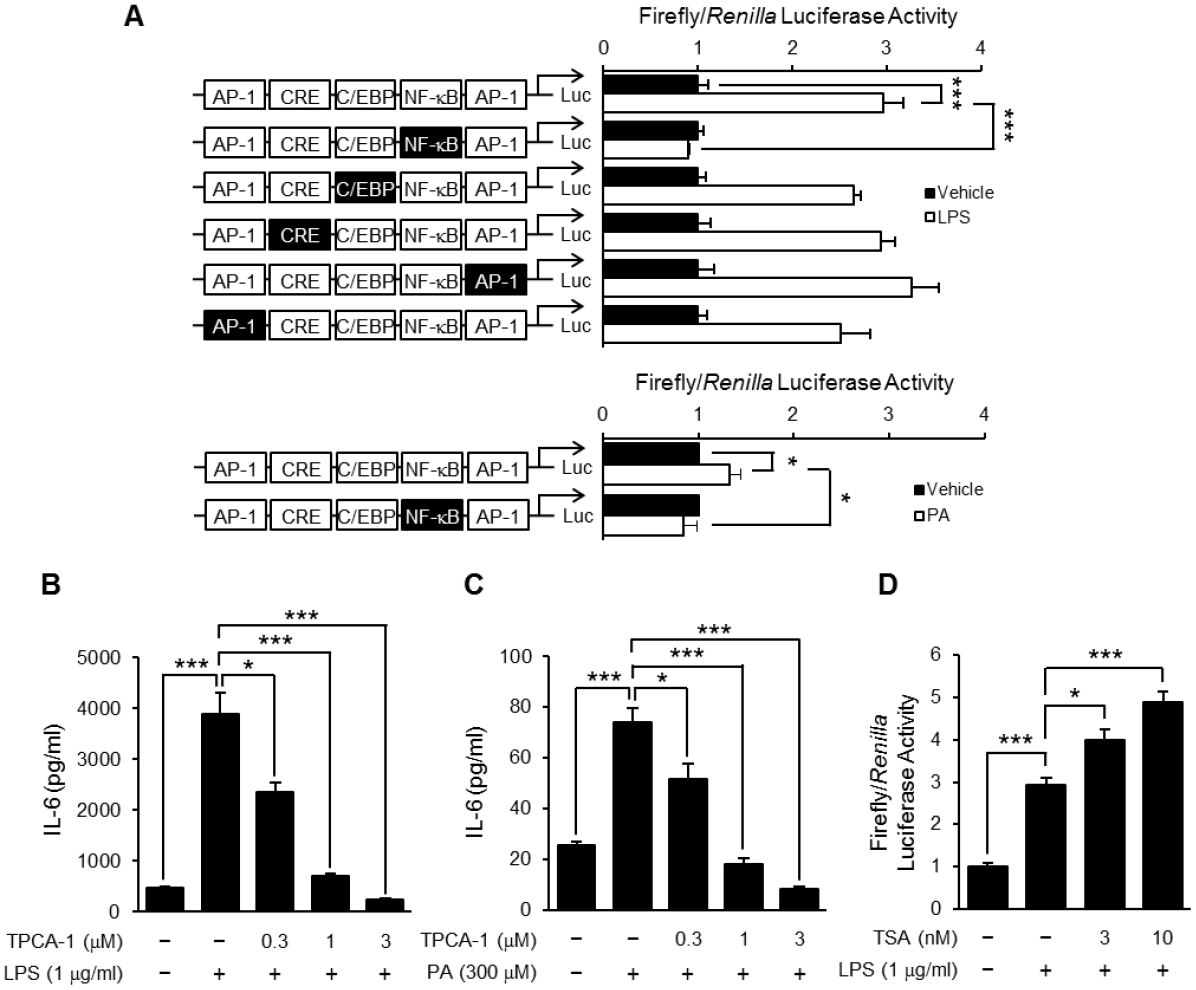
Effect of TSA on LPS-induced NF-κB-dependent transcriptional activation. OP9 preadipocytes were transiently transfected with luciferase gene constructs with a wild-type IL-6 promoter or that including a mutation in putative transcription factor-binding sites as indicated by black boxes, and then stimulated with LPS (1 µg/ml) or PA (300 µM). The luciferase activity in the cells cultured for 8 hours was determined (A). Values are normalized to those of *Renilla* luciferase activity and expressed as the fold-increase over the unstimulated control. Data represent the mean ± S.E.M. (*n* = 4). OP9 preadipocytes were preincubated for 1 hour in the presence or absence of TPCA-1, and then stimulated with LPS (1 µg/ml) (B) or PA (300 µM) (C). The concentration of IL-6 in the medium 24 hours after the stimulation was determined by ELISA. Data represent the mean ± S.E.M. (*n* = 4). OP9 preadipocytes were transiently transfected with the NF-κB-responsive luciferase reporter gene construct. The transfected cells were preincubated for 1 hour in the presence or absence of TSA, and then stimulated with LPS (1 µg/ml). The luciferase activity in the cells cultured for 8 hours was determined (D). Values are normalized to those of *Renilla* luciferase activity and expressed as the fold-increase over the unstimulated control. Data represent the mean ± S.E.M. (*n* = 4). ***P<0.001, *P<0.05 between the indicated groups.

Thirdly, we examined whether TSA affected the LPS-induced activation of transcription factors. To clarify which transcription factors contributed to IL-6 production in preadipocytes, a reporter gene assay was carried out using a wild-type or mutated IL-6 promoter construct, which has AP-1, NF-κB and C/EBP-binding sites and CRE. As shown in Fig. 7A, the transcriptional activity of the wild-type IL-6 promoter construct was increased by LPS or PA stimulation, and completely abolished by mutation of the NF-κB-binding site. In contrast, the mutation of the AP-1 and C/EBP-binding sites and CRE in the IL-6 promoter less affected transcriptional activity. These results were confirmed by using TPCA-1, an IKK-2 inhibitor. TPCA-1 pretreatment completely suppressed LPS- or PA-induced IL-6 production in a concentration-dependent manner (Fig. 7B and C). Therefore, we focused on LPS-induced transcriptional activation of NF-κB. As shown in Fig. 7D, stimulation with LPS increased NF-κB-dependent transcriptional activity and the effect was enhanced by TSA pretreatment.

### TSA increased acetylation of NF-κB p65 at lysine 310 and the duration of its nuclear translocation

**Figure 8:**
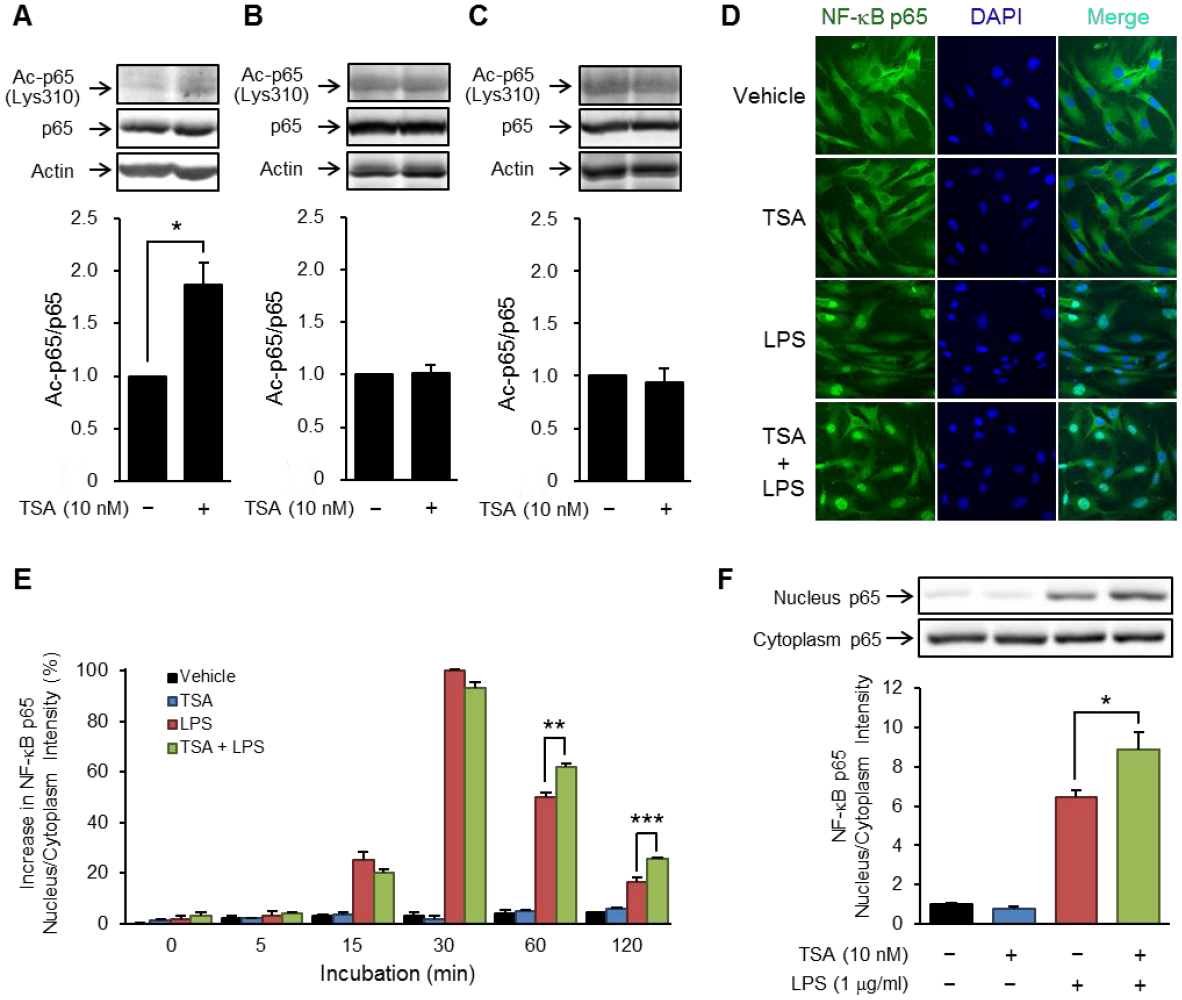
Effects of TSA on acetylation and LPS-induced nuclear translocation of NF-κB p65. OP9 preadipocytes (A) and mature adipocytes induced to differentiate by IO for 4 days (B) or TGZ for 6 days (C) were incubated for 1 hour with TSA (10 nM). The acetylated p65 was detected by Western blotting. The mean value at time 0 (vehicle) is set to 1.0. Data represent the mean ± S.E.M. (*n* = 3−5). OP9 preadipocytes were preincubated for 1 hour in the presence or absence of TSA (10 nM), and then stimulated with LPS (1 µg/ml) for the period indicated. p65 in the cells was immunostained (green) and the nucleus was labeled with DAPI (blue). A representative image of immunostaining of the cells stimulated for 60 minutes with LPS (D). The nuclear and cytoplasmic ratio of p65-associated fluorescence intensity is indicated as the average for 4 randomly selected fields (E). The values at time 0 (vehicle) and time 30 (LPS only) are set to 0 and 100, respectively. Data represent the mean ± S.E.M. (*n* = 4). The cells stimulated for 60 minutes with LPS were fractionated to cytoplasm and nucleus, and then p65 in these fractions was detected by Western blotting (F). The value for the control is set to 1.0. Data represent the mean ± S.E.M. (*n* = 4). ***P<0.001, **P<0.01, *P<0.05 between the indicated groups.

Because acetylation at lysine 310 increases the transcriptional activity of p65, we next examined the effects of TSA on this acetylation. As shown in Fig. 8A, B and C, acetylation of p65 at lysine 310 was increased 1 hour after the treatment with TSA in preadipocytes but not in mature adipocytes. The nuclear translocation of p65 was temporarily induced by LPS (Fig. 8D) and reached a peak at 30 minutes after LPS stimulation (Fig. 8E). Moreover, although the nuclear translocation of p65 at 0−30 minutes was less affected by TSA, that from 60 to 120 minutes was significantly enhanced (Fig. 8E). The enhancement was confirmed by the determination of levels of p65 in the nuclear and cytoplasmic fractions. At 60 minutes, LPS markedly increased the levels of p65 in the nuclear fraction and this was enhanced by TSA although TSA alone had no effect (Fig. 8F). These results suggested that the efflux of p65 was reduced by the treatment with TSA.

## Discussion

In the present study, we clarified that TSA, a HDAC inhibitor, enhanced the expression of inflammatory proteins and NF-κB-dependent transcriptional activity in OP9 preadipocytes. These results might be caused by increases in the acetylation of NF-κB p65 at lysine 310 and duration of the nuclear translocation of NF-κB. On the other hand, TSA did not affect LPS-induced IL-6 production and acetylation of NF-κB p65 at lysine 310 in mature adipocytes.

HDAC inhibitors did not themselves induce IL-6 production, but they increased LPS-induced IL-6 production in OP9 preadipocytes (Fig.3). The enhancement of TSA was not dependent on TLR4. Although TSA treatment increased the mRNA levels of TLR4 in embryonic stem cells and intestinal epithelial cells [35], [36], it did not do so in OP9 preadipocytes (Fig. 5A). In addition, TNFα-induced IL-6 production was also enhanced by TSA (Fig. 5B), indicating that TSA affected the expression and activities of signaling molecules and/or transcription factors.

Inflammatory stimulation can induce the phosphorylation of MAPKs, increasing their activity and the subsequent expression of inflammatory proteins. We therefore focused on MAPKs as candidates for proteins whose activity is affected by TSA. HDAC inhibitors affect the phosphorylation of MAPKs in several cell lines. For example, valproic acid induced the phosphorylation of p38 MAPK but not ERK1/2 or JNK in microglial cells and lymphocytes [37], [38]. However, we found that, in OP9 preadipocytes, the LPS-induced phosphorylation of ERK1/2 and p38 MAPK were not affected by TSA treatment whereas the production of IL-6 was reduced by a MEK inhibitor and a p38 MAPK inhibitor (Fig. 6).

We next focused on transcription factors including NF-κB, which is a key regulator for inflammatory protein expression. In fact, the experiments using reporter gene constructs containing a wild-type or mutated IL-6 promoter indicated that NF-κB strongly contributed to LPS- and PA-induced IL-6 production in preadipocytes (Fig. 7A). These results were confirmed by using an IKK-2 inhibitor (Fig. 7B and C). More importantly, we found that TSA enhanced LPS-induced NF-κB-dependent transcriptional activation in preadipocytes (Fig. 7D). Acetylation of the p65 subunit of NF-κB was shown to be critical to the regulation of NF-κB's activity by affecting transcriptional activity and the duration of nuclear translocation. The acetylation of p65 is regulated by HATs such as p300 and CBP and by HDACs, especially HDAC3 [39]–[42]. For example, acetylation of p65 at lysine 310 is associated with an increase in transcriptional activity without affecting the translocation into the nucleus. On the other hand, acetylation of p65 at lysine 218 or 221 leads to an increase in the duration of nuclear translocation of NF-κB by attenuating its interaction with IκB. We clarified that both the acetylation of p65 at lysine 310 and the LPS-induced translocation of NF-κB into the nucleus were enhanced by treatment with TSA (Fig. 8). These results suggested that the increase in the nuclear translocation and transcriptional activity of NF-κB contribute to a rise in NF-κB-dependent transcriptional activity and subsequent expression of inflammatory proteins.

The expression of COX-2, iNOS and MCP-1, all enhanced by TSA as described in Fig. 4, was regulated by NF-κB but other transcription factors also regulate them separately. For example, the expression of MCP-1 is regulated by Sp1 in addition to NF-κB [43]. Because acetylation of Sp1 is associated with a decrease in its DNA-binding activity [44], TSA might induce hyperacetylation of Sp1 and act negatively on LPS-induced expression. Thus, modulation of the acetylation of transcription factors other than NF-κB was also involved in the HDAC inhibitor-induced enhancement of gene expression. In the future, there is a need to investigate the effects of HDAC inhibitors on the activity of transcription factors other than NF-κB in pre/mature adipocytes.

The OP9 cell line was established from the calvaria of newborn mice genetically deficient in functional macrophage colony-stimulating factor [45], and is used as a pre/mature adipocyte model [28]. It is known that, during adipocyte differentiation, the expression of various proteins including transcription factors changes dynamically [28], [46]. Notably, an increase in PPARγ induces the gene expression of several adipocyte-specific proteins (ex. adiponectin) and other transcription factors (ex. C/EBPα) thus transforming the cell into the characteristic lipid-rich mature adipocyte [47]. Based on the results in Fig. 1, we selected an incubation period of 4 days for the IO-based method and 6 days for the TGZ-based method, as appropriate for differentiation. Interestingly, the enhancing effects of TSA on LPS-induced IL-6 production were observed in preadipocytes but not mature adipocytes (Fig. 2 and 3). Treatment with TSA increased acetylation of p65 at lysine 310 in preadipocytes but not mature adipocytes (Fig. 8A, B and C), which might be one cause of the difference in the effects of HDAC inhibitors. In adult humans, preadipocytes account for 20−40% of cells in white adipose tissue [48] and produce inflammatory proteins via TLR4 signaling as well as infiltrated macrophages [49]–[51]. Moreover, LPS-induced IL-6 production was much lower in mature adipocytes than preadipocytes [50], [51]. These findings indicate that preadipocytes play a greater role in chronic inflammation in adipose tissue than do mature adipocytes.

Some reports have shown that HDAC inhibitors suppressed inflammatory responses in macrophages at relatively high concentrations [27], [52], [53]. We also confirmed that TSA and SAHA decreased LPS-induced IL-6 production in RAW264 macrophages (data not shown). The proposed mechanism by which HDAC inhibitors exert their effects is interference with RNA polymerase II's recruitment to inflammatory genes [54]. In contrast, in microglial cells, HDAC inhibitors enhanced the production of inflammatory proteins [24] as consistent with our findings in preadipocytes. Thus HDAC inhibitors exert negative and positive effects on the expression of inflammatory proteins probably dependent on types of cells and on concentrations. The precise mechanisms remained to be elucidated.

The enhancement of LPS- and PA-induced inflammatory protein expression in preadipocytes is important to the maintenance of glucose homeostasis. Chronically elevated IL-6 levels in obesity have been considered to contribute to systemic insulin resistance by inducing the expression of suppressor of cytokine signaling 3 in adipose tissue and liver [7], [55], [56]. In contrast, it was recently reported that IL-6, especially produced temporarily in response to acute stimuli such as exercise, improved glucose tolerance in skeletal muscle, liver and intestinal L cells [57]–[59]. Thus the roles of IL-6 in glucose metabolism might differ with the duration and extent of IL-6 production.

In conclusion, we clarified that HDAC inhibitors enhanced the production of inflammatory cytokines in preadipocytes but not in adipocytes by affecting NF-κB through acetylation of the p65 subunit. These findings shed new light on the roles of HDACs in preadipocytes in the production of inflammatory proteins and on the regulation of systemic glucose homeostasis.

## References

[1] Xu H, Barnes GT, Yang Q, Tan G, Yang D, et al (2003) Chronic inflammation in fat plays a crucial role in the development of obesity-related insulin resistance. J Clin Invest 112: 1821–1830. 14679177 10.1172/JCI19451 PMC296998

[2] Southern C, Schulster D, Green IC (1990) Inhibition of Insulin-Secretion from Rat Islets of Langerhans by Interleukin-6 - an Effect Distinct from That of Interleukin-1. Biochem J 272: 243–245. 2264829 10.1042/bj2720243 PMC1149683

[3] Senn JJ, Klover PJ, Nowak IA, Mooney RA (2002) Interleukin-6 induces cellular insulin resistance in hepatocytes. Diabetes 51: 3391–3399. 12453891 10.2337/diabetes.51.12.3391

[4] Rotter V, Nagaev I, Smith U (2003) Interleukin-6 (IL-6) induces insulin resistance in 3T3-L1 adipocytes and is, like IL-8 and tumor necrosis factor-alpha, overexpressed in human fat cells from insulin-resistant subjects. J Biol Chem 278: 45777–45784. 12952969 10.1074/jbc.M301977200

[5] Senn JJ, Klover PJ, Nowak IA, Zimmers TA, Koniaris LG, et al (2003) Suppressor of cytokine signaling-3 (SOCS-3), a potential mediator of interleukin-6-dependent insulin resistance in hepatocytes. J Biol Chem 278: 13740–13746. 12560330 10.1074/jbc.M210689200

[6] Klover PJ, Zimmers TA, Koniaris LG, Mooney RA (2003) Chronic exposure to interleukin-6 causes hepatic insulin resistance in mice. Diabetes 52: 2784–2789. 14578297 10.2337/diabetes.52.11.2784

[7] Klover PJ, Clementi AH, Mooney RA (2005) Interleukin-6 depletion selectively improves hepatic insulin action in obesity. Endocrinology 146: 3417–3427. 15845623 10.1210/en.2004-1468

[8] Kanda H, Tateya S, Tamori Y, Kotani K, Hiasa K, et al (2006) MCP-1 contributes to macrophage infiltration into adipose tissue, insulin resistance, and hepatic steatosis in obesity. J Clin Invest 116: 1494–1505. 16691291 10.1172/JCI26498 PMC1459069

[9] Beutler B, Rietschel ET (2003) Innate immune sensing and its roots: the story of endotoxin. Nat Rev Immunol 3: 169–176. 12563300 10.1038/nri1004

[10] Park BS, Song DH, Kim HM, Choi BS, Lee H, et al (2009) The structural basis of lipopolysaccharide recognition by the TLR4-MD-2 complex. Nature 458: 1191–1195. 19252480 10.1038/nature07830

[11] Cani PD, Amar J, Iglesias MA, Poggi M, Knauf C, et al (2007) Metabolic endotoxemia initiates obesity and insulin resistance. Diabetes 56: 1761–1772. 17456850 10.2337/db06-1491

[12] Shi H, Kokoeva MV, Inouye K, Tzameli I, Yin H, et al (2006) TLR4 links innate immunity and fatty acid-induced insulin resistance. J Clin Invest 116: 3015–3025. 17053832 10.1172/JCI28898 PMC1616196

[13] Eguchi K, Manabe I, Oishi-Tanaka Y, Ohsugi M, Kono N, et al (2012) Saturated fatty acid and TLR signaling link beta cell dysfunction and islet inflammation. Cell Metab 15: 518–533. 22465073 10.1016/j.cmet.2012.01.023

[14] Regnier CH, Song HY, Gao X, Goeddel DV, Cao Z, et al (1997) Identification and characterization of an IkappaB kinase. Cell 90: 373–383. 9244310 10.1016/s0092-8674(00)80344-x

[15] Jacobs MD, Harrison SC (1998) Structure of an IkappaBalpha/NF-kappaB complex. Cell 95: 749–758. 9865693 10.1016/s0092-8674(00)81698-0

[16] Struhl K (1998) Histone acetylation and transcriptional regulatory mechanisms. Genes Dev 12: 599–606. 9499396 10.1101/gad.12.5.599

[17] Strahl BD, Allis CD (2000) The language of covalent histone modifications. Nature 403: 41–45. 10638745 10.1038/47412

[18] Glozak MA, Sengupta N, Zhang X, Seto E (2005) Acetylation and deacetylation of non-histone proteins. Gene 363: 15–23. 16289629 10.1016/j.gene.2005.09.010

[19] Khan N, Jeffers M, Kumar S, Hackett C, Boldog F, et al (2008) Determination of the class and isoform selectivity of small-molecule histone deacetylase inhibitors. Biochem J 409: 581–589. 17868033 10.1042/BJ20070779

[20] Blanchard F, Chipoy C (2005) Histone deacetylase inhibitors: new drugs for the treatment of inflammatory diseases? Drug Discov Today 10: 197–204. 15708534 10.1016/S1359-6446(04)03309-4

[21] Ito K, Barnes PJ, Adcock IM (2000) Glucocorticoid receptor recruitment of histone deacetylase 2 inhibits interleukin-1beta-induced histone H4 acetylation on lysines 8 and 12. Mol Cell Biol 20: 6891–6903. 10958685 10.1128/mcb.20.18.6891-6903.2000 PMC88765

[22] Ashburner BP, Westerheide SD, Baldwin AS Jr (2001) The p65 (RelA) subunit of NF-kappaB interacts with the histone deacetylase (HDAC) corepressors HDAC1 and HDAC2 to negatively regulate gene expression. Mol Cell Biol 21: 7065–7077. 11564889 10.1128/MCB.21.20.7065-7077.2001 PMC99882

[23] Zhong H, May MJ, Jimi E, Ghosh S (2002) The phosphorylation status of nuclear NF-kappa B determines its association with CBP/p300 or HDAC-1. Mol Cell 9: 625–636. 11931769 10.1016/s1097-2765(02)00477-x

[24] Suuronen T, Huuskonen J, Pihlaja R, Kyrylenko S, Salminen A (2003) Regulation of microglial inflammatory response by histone deacetylase inhibitors. J Neurochem 87: 407–416. 14511118 10.1046/j.1471-4159.2003.02004.x

[25] Miao F, Gonzalo IG, Lanting L, Natarajan R (2004) In vivo chromatin remodeling events leading to inflammatory gene transcription under diabetic conditions. J Biol Chem 279: 18091–18097. 14976218 10.1074/jbc.M311786200

[26] Ito K, Yamamura S, Essilfie-Quaye S, Cosio B, Ito M, et al (2006) Histone deacetylase 2-mediated deacetylation of the glucocorticoid receptor enables NF-kappaB suppression. J Exp Med 203: 7–13. 16380507 10.1084/jem.20050466 PMC2118081

[27] Yu Z (2002) Histone Deacetylases Augment Cytokine Induction of the iNOS Gene. J Am Soc Nephrol 13: 2009–2017. 12138131 10.1097/01.asn.0000024253.59665.f1

[28] Wolins NE, Quaynor BK, Skinner JR, Tzekov A, Park C, et al (2006) OP9 mouse stromal cells rapidly differentiate into adipocytes: characterization of a useful new model of adipogenesis. J Lipid Res 47: 450–460. 16319419 10.1194/jlr.D500037-JLR200

[29] Davies JM, MacSharry J, Shanahan F (2010) Differential regulation of Toll-like receptor signalling in spleen and Peyer's patch dendritic cells. Immunology 131: 438–448. 20545785 10.1111/j.1365-2567.2010.03317.x PMC2996564

[30] Gonzales AM, Orlando RA (2008) Curcumin and resveratrol inhibit nuclear factor-kappaB-mediated cytokine expression in adipocytes. Nutr Metab (Lond) 5: 17. 18549505 10.1186/1743-7075-5-17 PMC2441623

[31] Tao JY, Zheng GH, Zhao L, Wu JG, Zhang XY, et al (2009) Anti-inflammatory effects of ethyl acetate fraction from Melilotus suaveolens Ledeb on LPS-stimulated RAW 264.7 cells. J Ethnopharmacol 123: 97–105. 19429346 10.1016/j.jep.2009.02.024

[32] Rhinn H, Marchand-Leroux C, Croci N, Plotkine M, Scherman D, et al (2008) Housekeeping while brain's storming Validation of normalizing factors for gene expression studies in a murine model of traumatic brain injury. BMC Mol Biol 9: 62. 18611280 10.1186/1471-2199-9-62 PMC2500043

[33] Hirasawa N, Yashima K, Ishihara K (2009) Enhancement of ligand-dependent down-regulation of glucocorticoid receptor by lipopolysaccharide. Life Sci 85: 578–585. 19729027 10.1016/j.lfs.2009.08.012

[34] Baccam M, Woo SY, Vinson C, Bishop GA (2003) CD40-mediated transcriptional regulation of the IL-6 gene in B lymphocytes: involvement of NF-kappa B, AP-1, and C/EBP. J Immunol 170: 3099–3108. 12626566 10.4049/jimmunol.170.6.3099

[35] Zampetaki A, Xiao Q, Zeng L, Hu Y, Xu Q (2006) TLR4 expression in mouse embryonic stem cells and in stem cell-derived vascular cells is regulated by epigenetic modifications. Biochem Biophys Res Commun 347: 89–99. 16814255 10.1016/j.bbrc.2006.06.055

[36] Takahashi K, Sugi Y, Hosono A, Kaminogawa S (2009) Epigenetic regulation of TLR4 gene expression in intestinal epithelial cells for the maintenance of intestinal homeostasis. J Immunol 183: 6522–6529. 19846881 10.4049/jimmunol.0901271

[37] Xie N, Wang C, Lin Y, Li H, Chen L, et al (2010) The role of p38 MAPK in valproic acid induced microglia apoptosis. Neurosci Lett 482: 51–56. 20621161 10.1016/j.neulet.2010.07.004

[38] Chen Q, Ouyang DY, Geng M, Xu LH, Zhang YT, et al (2011) Valproic acid exhibits biphasic effects on apoptotic cell death of activated lymphocytes through differential modulation of multiple signaling pathways. J Immunotoxicol 8: 210–218. 21457087 10.3109/1547691X.2011.568979

[39] Chen L, Fischle W, Verdin E, Greene WC (2001) Duration of nuclear NF-kappaB action regulated by reversible acetylation. Science 293: 1653–1657. 11533489 10.1126/science.1062374

[40] Chen LF, Mu Y, Greene WC (2002) Acetylation of RelA at discrete sites regulates distinct nuclear functions of NF-kappaB. EMBO J 21: 6539–6548. 12456660 10.1093/emboj/cdf660 PMC136963

[41] Chen LF, Greene WC (2004) Shaping the nuclear action of NF-kappaB. Nat Rev Mol Cell Biol 5: 392–401. 15122352 10.1038/nrm1368

[42] Ghizzoni M, Haisma HJ, Maarsingh H, Dekker FJ (2011) Histone acetyltransferases are crucial regulators in NF-kappaB mediated inflammation. Drug Discov Today 16: 504–511. 21477662 10.1016/j.drudis.2011.03.009 PMC5218544

[43] Ping D, Boekhoudt GH, Rogers EM, Boss JM (1999) Nuclear factor-kappa B p65 mediates the assembly and activation of the TNF-responsive element of the murine monocyte chemoattractant-1 gene. J Immunol 162: 727–734. 9916692

[44] Waby JS, Chirakkal H, Yu C, Griffiths GJ, Benson RS, et al (2010) Sp1 acetylation is associated with loss of DNA binding at promoters associated with cell cycle arrest and cell death in a colon cell line. Mol Cancer 9: 275. 20950428 10.1186/1476-4598-9-275 PMC2972244

[45] Nakano T, Kodama H, Honjo T (1994) Generation of lymphohematopoietic cells from embryonic stem cells in culture. Science 265: 1098–1101. 8066449 10.1126/science.8066449

[46] Rosen ED, Walkey CJ, Puigserver P, Spiegelman BM (2000) Transcriptional regulation of adipogenesis. Genes Dev 14: 1293–1307. 10837022

[47] Tontonoz P, Hu E, Graves RA, Budavari AI, Spiegelman BM (1994) mPPAR gamma 2: tissue-specific regulator of an adipocyte enhancer. Genes Dev 8: 1224–1234. 7926726 10.1101/gad.8.10.1224

[48] Hauner H (2005) Secretory factors from human adipose tissue and their functional role. Proc Nutr Soc 64: 163–169. 15960861 10.1079/pns2005428

[49] Fried SK, Bunkin DA, Greenberg AS (1998) Omental and subcutaneous adipose tissues of obese subjects release interleukin-6: depot difference and regulation by glucocorticoid. J Clin Endocrinol Metab 83: 847–850. 9506738 10.1210/jcem.83.3.4660

[50] Harkins JM, Moustaid-Moussa N, Chung YJ, Penner KM, Pestka JJ, et al (2004) Expression of interleukin-6 is greater in preadipocytes than in adipocytes of 3T3-L1 cells and C57BL/6J and ob/ob mice. J Nutr 134: 2673–2677. 15465765 10.1093/jn/134.10.2673

[51] Chung S, Lapoint K, Martinez K, Kennedy A, Boysen Sandberg M, et al (2006) Preadipocytes mediate lipopolysaccharide-induced inflammation and insulin resistance in primary cultures of newly differentiated human adipocytes. Endocrinology 147: 5340–5351. 16873530 10.1210/en.2006-0536

[52] Grabiec AM, Korchynskyi O, Tak PP, Reedquist KA (2012) Histone deacetylase inhibitors suppress rheumatoid arthritis fibroblast-like synoviocyte and macrophage IL-6 production by accelerating mRNA decay. Ann Rheum Dis 71: 424–431. 21953341 10.1136/ard.2011.154211 PMC3277722

[53] Choi Y, Park SK, Kim HM, Kang JS, Yoon YD, et al (2008) Histone deacetylase inhibitor KBH-A42 inhibits cytokine production in RAW 264.7 macrophage cells and in vivo endotoxemia model. Exp Mol Med 40: 574–581. 18985016 10.3858/emm.2008.40.5.574 PMC2679350

[54] Furumai R, Ito A, Ogawa K, Maeda S, Saito A, et al (2011) Histone deacetylase inhibitors block nuclear factor-kappaB-dependent transcription by interfering with RNA polymerase II recruitment. Cancer Sci 102: 1081–1087. 21299717 10.1111/j.1349-7006.2011.01904.x

[55] Sabio G, Das M, Mora A, Zhang Z, Jun JY, et al (2008) A stress signaling pathway in adipose tissue regulates hepatic insulin resistance. Science 322: 1539–1543. 19056984 10.1126/science.1160794 PMC2643026

[56] Matsubara T, Mita A, Minami K, Hosooka T, Kitazawa S, et al (2012) PGRN is a key adipokine mediating high fat diet-induced insulin resistance and obesity through IL-6 in adipose tissue. Cell Metab 15: 38–50. 22225875 10.1016/j.cmet.2011.12.002

[57] Pedersen BK, Febbraio MA (2008) Muscle as an endocrine organ: focus on muscle-derived interleukin-6. Physiol Rev 88: 1379–1406. 18923185 10.1152/physrev.90100.2007

[58] Awazawa M, Ueki K, Inabe K, Yamauchi T, Kubota N, et al (2011) Adiponectin enhances insulin sensitivity by increasing hepatic IRS-2 expression via a macrophage-derived IL-6-dependent pathway. Cell Metab 13: 401–412. 21459325 10.1016/j.cmet.2011.02.010

[59] Ellingsgaard H, Hauselmann I, Schuler B, Habib AM, Baggio LL, et al (2011) Interleukin-6 enhances insulin secretion by increasing glucagon-like peptide-1 secretion from L cells and alpha cells. Nat Med 17: 1481–1489. 22037645 10.1038/nm.2513 PMC4286294

